# ABA signal in rice under stress conditions

**DOI:** 10.1186/1939-8433-5-1

**Published:** 2012-02-27

**Authors:** Nenghui Ye, Liguo Jia, Jianhua Zhang

**Affiliations:** 1 Department of Biology, Hong Kong Baptist University, Hong Kong, China; 2 School of Life Sciences and State Key Laboratory of Agrobiotechnology, The Chinese University of Hong Kong, Hong Kong, China

**Keywords:** abscisic acid (ABA), ABA transporter, ABA receptor, ABA signaling, ABA metabolism, ABA biosynthesis, abiotic and biotic stresses, rice (*Oryza sativa*)

## Abstract

Ever since its discovery, abscisic acid (ABA) has been intensively studied due to its versatile functions in plant developmental and physiological processes. Many signaling details of ABA have been well elucidated and reviewed. The identification of ABA receptors is a great breakthrough in the field of ABA study, whereas the discovery of ABA transporter has changed our concept that ABA is delivered solely by passive transport. The intensity of ABA signaling pathway is well known to be controlled by multi-regulators. Nonetheless, the interaction and coordination among ABA biosynthesis, catabolism, conjugation and transportation are seldom discussed. Here, we summarize the biological functions of ABA in response to different stresses, especially the roles of ABA in plant defense to pathogen attack, and discuss the possible relationships of these determinants in controlling the specificity and intensity of ABA signaling pathway in the rice.

## Introduction

Abscisic acid (ABA) is a plant stress hormone and one of the foremost important signaling molecules in plants, which plays versatile functions in regulating many developmental processes and adaptive stress processes (Santner *et al*., 2009; Cutler *et al*., 2010). The signaling pathways of ABA is intensively studied ever since its discovery and great progresses have been made after several decades of studies (Hirayama and Shinozaki, 2007; Wasilewska *et al*., 2008). ABA is famous for its stress-related properties but it also has been proven to regulate many development and growth processes. Over the past 30 years' research, the network of ABA signaling which is extremely complicated has been gradually elucidated by forward and reverse genetic, biochemical and pharmacological methods, in which ABA acts as a "hub" or even a "free agent" (Finkelstein and Gibson, 2002; Culter *et al*., 2010; Raghavendra *et al*., 2010). In recent years, the identification of ABA receptors in plants is an exciting breakthrough in this field and has further completed this complicated network (Ma *et al*., 2009; Park *et al*., 2009; Weiner *et al*., 2010). The molecular mechanism and parts of abiotic-related functions of ABA signaling pathway have been well discussed in some outstanding reviews (Cutler *et al*., 2010; Raghavendra *et al*., 2010). Therefore, in this review, we focus on the recent progress made in revealing functions of ABA signaling in an integrated level and highlight how the homeostasis of this chemical is controlled by plants in response to stresses.

## The metabolism of ABA in rice under stresses

Like other plant hormones, concentration of ABA in the tissue as well as the sensitivity of the tissue to ABA will determine what response to this phytohormone. The concentration of ABA in a specific tissue is also determined by the process of biosynthesis, catabolism, compartmentation and transport (Taiz and zeiger, 2006). To date, the ABA biosynthesis and catabolism pathway has been completed by using different mutant of these pathways, genetic approaches and other feeding experiments.

## ABA biosynthesis and its regulating genes in rice

Biosynthesis of ABA in higher plants is followed in an 'indirect' pathway which begins with isopentenyl pyrophosphate (IPP), the biological isoprene unit. IPP is the precursor of all terpenoids as well as many plant hormones. A more specific pathway to ABA biosynthesis starts from the epoxidation of zeaxanthin and antheraxanthin to violaxanthin, which occurs in chloroplasts and other plastids and is catalyzed by a zeaxanthin epoxidase (ZEP) (Agrawal *et al*., 2001). The subsequent conversion to 9-*cis*-epoxycarotenoid, the *cis-*isomers of violaxanthin and neoxanthin, is catalyzed by the enzyme ABA4 which is the latest solved by position cloning of the *ABA4 *gene (North *et al*., 2007). Both *cis-*violaxanthin and *cis-*neoxanthin are alternative substrate of *9-cis-*epoxycarotenoid dioxygenase (NCED) and oxidative cleavage of them leads to the production of xanthoxin--the first cytoplasmic precursor for the catalytic conversion to ABA (Schwartz *et al*., 2003). In the cytoplasm, a two-step reaction via ABA-aldehyde takes place, which convert xanthoxin into ABA. The first step in cytoplasm that converts xanthoxin into ABA-aldehyde is catalyzed by ABA2 which belongs to the short-chain dehydrogenase/redutase (SDR) family (Cheng *et al*., 2002). ABA aldehyde oxidase (AAO) with molybdenum cofactor (MoCo) as cofactor then catalyzes ABA-aldehyde into ABA, which is the last step of ABA biosynthesis pathway (Seo *et al*., 2004).

Among the members of ABA biosynthesis enzymes, NCED is the foremost one which catalyzes the regulating step of this pathway. The first *NCED *gene (*VP14*) was cloned in maize by insertional mutagenesis, and exhibits mild ABA-deficient phenotypes due to gene redundancy (Tan *et al*., 1997). To date, 5 NCED genes are most probably involved in ABA biosynthesis in *Arabidopsis *(Iuchi *et al*., 2000; Schwartz *et al*., 2003) and rice (Saika *et al*., 2007; Zhu *et al*., 2009). As is the case for other carotenoid biosynthesis enzymes, NCED proteins from various species are located in chloroplast (Qin and Zeevaart, 1999; Iuchi *et al*., 2001; Tan *et al*., 2003). Gene expression analysis reveals that the transcript level of *OsNCED2 *gene is abundant in seed (Zhu *et al*., 2009) while *OsNCED1 *is mainly expressed in rice leaves (Ye *et al*., 2011). Like *Arabidopsis*, the *NCED3 *gene is significantly induced by water stress, which is responsible for the dramatically increase of ABA level in rice and *Arabidopsis *exposed to water stress (Tan *et al*., 2003; Ye *et al*., 2011). NCED3 is also reported to express in root, indicating its function in responding to environmental cues (Tan *et al*., 2003). Interestingly, the *OsNCED1 *gene, which has the highest expression level in rice leaf and is the housekeeping gene in normal conditions, is significantly suppressed by water stress. This may lead to the conclusion that ABA accumulation has a feedback effect on the expression of *OsNCED1 *gene (Tian *et al*. 2004).

## ABA catabolism and the committed step

For ABA action, there must be an accumulation of biologically active ABA at the site of perception. Although ABA synthesis is required, whether or not it is the main factor in controlling how much ABA accumulates under stress is unclear. Indeed, it is possible that the content of phaseic acid (PA), the principal catabolite of ABA, accumulates significantly whereas ABA content does not have any distinct increase although the biosynthesis key gene *NCED3 *is evidently induced by the stresses (Qin and Zeewaart, 2002; Priest *et al*., 2006). Application of exogenous ABA can also induce PA accumulation (Huang *et al*., 2007). Besides, other labeling and inhibiting experiments have further proved a rapid turnover of ABA is essential for plants under both normal and stress conditions (Cornish and Zeewaart, 1984; Ribaut *et al*., 1996). Their results all suggest that ABA synthesis and catabolism are determinants for ABA concentration in plants under stress conditions.

When compared with ABA biosynthesis pathway, ABA catabolism is much simpler. ABA can be hydroxylated at three different methyl groups in the ring structure (C-7', C-8', and C-9'), which leads to three pathways for ABA hydroxylation and produces three substantial biological activities metabolites (Zhou *et al*., 2004). Although the hydroxylation does not reduce the biological activity of ABA thoroughly, it can trigger further inactivation steps. Among the hydroxylated products, only the 8'-hydroxy ABA can be changed into PA by cyclization and then into dihydophaseic acid (DPA) by further reduction (Nambara and Marion-Poll, 2005). DPA is the end product of ABA catabolism which does not exhibit any ABA-like activity in any of the standard protocols. Besides, the C-8' hydroxylation is commonly thought to be the predominant ABA catabolic pathway and as a result, PA and DPA are the most widespread and abundant ABA catabolites (Culter and Krochko, 1999).

From ABA to DPA, there are only three steps. The first step of ABA catabolism has been proven to be the committed step, which is mainly catalyzed by *CYP707A *gene in *Arabidopsis *(Kushiro *et al*., 2004; Saito *et al*., 2004) and *OsABA8ox *gene in rice (Yang and Choi, 2006). The *CYP707A *gene has been proven to hydroxylate ABA but is not involved in cyclization of 8'-hydroxy ABA to PA (Saito *et al*., 2004). There are 4 *CYP707A *genes in *Arabidopsis *and *CYP707A1 *and *CYP707A3 *genes are abundant in most tissue of *Arabidopsis *(Saito *et al*., 2004). In rice, there are 3 ABA 8'-hydroxylases identified (Saika *et al*., 2007). According to their expression analysis, both *OsABA8ox2 *and *OsABA8ox3 *gene are induced significantly early in seed germination and are responsible for the decrease of ABA level during seed germination. Both of their expressions, especially *OsABA8ox3 *gene, are suppressed by glucose which can delay seed germination (Zhu *et al*., 2009). Whereas in rice leaves, it is the *OsABA8ox1 *gene that is dramatically induced by rehydration, which can finally lead to the decrease of ABA content in rice leaf (Ye *et al*., 2011). These results indicate that different ABA 8'-hydroxylases functions in different tissues and various development processes. Also ABA catabolism is important in contribute to the ABA concentration in plant under non-stress or stress conditions.

## ABA conjugation and deconjugation

Except ABA biosynthesis and catabolism, ABA conjugation has been recently reported to play key role in *Arabidopsis *and acts as a quick mechanism in response to abiotic stresses (Lee *et al*., 2006; Wasilewska *et al*., 2008). ABA is inactivated at the C-1 hydroxyl group by different chemical which form different conjugates and accumulate in vacuoles or apoplastic space (Lehmann and Glund, 1986; Dietz *et al*., 2000). Among them, the ABA glucosyl ester (ABA-GE) is the most widespread conjugate which is catalyzed by ABA glucosyltransferase (Boyer and Zeevaart, 1982; Xu *et al*., 2002). The gene encoding ABA glucosyltransferase was firstly identified in adzuki bean and named AOG (Xu *et al*., 2002). The recombinant AOG protein was reported to conjugate ABA with UDP-D-glucose. In *Arabidopsis*, eight glycosylases were identified by using screening strategy. Priest et al. (2006) further proved that one of them, UGT71B6, is involved in keeping the homeostasis in *Arabidopsis*. However, AOG from other species is seldom cloned owing to its broad substrate specificity. More effort is needed to further assess the importance of ABA conjugation in determining the ABA concentration in the acting site of plants (Wasilewska *et al*., 2008).

Like the ABA glucosyltransferase, the protein that release ABA from ABA-GE is still a mystery not until the identification of AtBG1 protein (Lee *et al*., 2006). Their inspiring results show that ABA conjugation also plays a pivotal role as an active ABA pool for plants to adjust to changing physiological and environmental conditions (Lee *et al*., 2006). The AtBG1, a β-glucosidase, was identified to hydrolyze ABA-GE to produce bioactive ABA. *Atbg1 *mutant lines exhibit lower ABA level and ABA defective phenotypes whereas over-expression lines increase levels of dehydration- induced ABA accumulation. The permeability of biomembranes for ABA-GE is very low, which makes ABA-GE well suitable for long-distance translocation and stored in vacuoles or apoplastic space (Dietz *et al*., 2000; Jiang and Hartung, 2007). The vacuolar or apoplastic ABA will then be transported by an unknown mechanism into the endoplasmic reticulum where it is cleaved to release bioactive ABA (Lee *et al*., 2006). Further biochemical study shows that the β-glucosidase in WT Arabidopsis under water stress is much higher than that in *Arabidopsis *in normal condition, indicating the importance of ABA conjugation and deconjugation in controlling the ABA levels in plants response to stress and that ABA conjugation is a dynamic process which may also play a key role, except for ABA biosynthesis and ABA catabolism, in determining the precise ABA concentration in the acting site (Verslues and Zhu, 2007).

## ABA transporter

When the plants are applied with ABA treatment on their roots, an elevated ABA content in leaves can be detected quickly from the onset of ABA application (Ye *et al*., 2011), indicating an efficient transport system for ABA in plants. ABA transport is historically assumed to be a diffusive process due to its permeable property to the cell membrane (Salisbury and Ross, 1985). However, when compared with auxin, another plant hormone known to be transported over long distance by an intricately mechanism, the transport for ABA should not be so simple. Indeed, many investigations have suggested that ABA transport should not occur solely by a diffusive process (Daeter and Hartung, 1993; Wilkinson and Davies, 1997; Jiang and Hartung, 2008). Unfortunately, little is known about the field of ABA transporter although many investigations have been done in searching for an ABA transporter for decades.

The discovery of *AtMRP5 *gene is involved in guard cell hormone signaling and water use has drawn the attention to ABC transporter family, an ATP-binding cassette transporter. Although stomata apertures in *atmrp5 *mutant are identical with wild type in the dark, the opening of stomata apertures is reduced in light compared with wide type plants. Foremost, the mutant is also insensitive to application of ABA, indicating the function of AtMRP5 in transporting ABA (Klein *et al*., 2003). Based on their results, many investigators have searched the ABC transporter family as candidate for ABA transporter. Recent years, two ABC transporters, PDR12/ABCG40 and AtABCG25, were identified to be ABA transporter. The *AtABC40 *is broadly expressed and its product locates in cell membrane (Kang *et al*., 2010). Uptake of ABA into yeast and BY2 cell expressing AtABCG40 increases compared with those without AtABCG40, whereas ABA uptake into protoplasts of *abcg40 *mutant plant is reduced. Gene expression in response to exogenous ABA is also delayed in *abcg40 *mutant, indicating that ABA transport is also involved in ABA-dependent signaling. Simultaneously, another publication from the same volume of the journal reported another ABA transporter, the AtABCG25 (Kuromori *et al*., 2010). Although AtABCG25 is also located in membrane, it is only abundant in vacuolar tissue, where ABA is synthesized. The membrane vesicles from insect cells which expressing *AtABCG25 *gene exhibited ABA transport. Likewise, in plant overexpressing the same gene showed a regulation on stomata movement, overexpression of *AtABCG25 *in *Arabidopsis *led to less transpiration from the leaves. Water loss from detached leaves of the transgenic plants was also slower than that from detached wild-type leaves, probably because this transporter facilitates the delivery of ABA to guard cells. These results indicate AtABCG25 is an exporter of ABA and is involved in the intercellular ABA signaling pathway. More recently, Kuromori et al. (2011) reported *AtABCG22*, the closest gene of *AtABCG25*, appear to code for an ABA importer. The *AtABCG22 *gene is expressed in aerial organs, mainly in guard cell. Mutation of this gene displays an increased water transpiration and drought susceptibility. Double mutant of *atabcg22 *with other genes shows that *atabcg22 *can enhance the phenotypes of *srk2e/ost1 *and *nced3 *mutants, which are defective in ABA signaling and biosynthesis, respectively.

The property of long-distance movement of ABA has made it a critical signal massager for plant in many developmental processes and in response to different abiotic stresses. Under conditions of mild stress as the soil starts to dry, when the water potential of the leaves is not or only slightly affected, ABA accumulates in root tissue and then release to the xylem vessels where ABA is transport to the acting site in the shoot (Zhang and Davies, 1990; Davies *et al*., 2005). Movement of ABA into plant cells has long been understood to occur via pH-dependent diffusion, such that when the pH of the xylem sap is increased by water stress (Wilkinson and Davies, 1997; Bahrun et al., 2002), there is a reduction of ABA movement into cells so that more ABA is available for mass transport within the transcription stream to sites of action in the shoot (Wilkinson and Davies, 1997). The identifications of ABA transporters in target cell membranes (e.g. guard cell) have resolved the issue of how ABA gains entry to its sites of action under condition in which the diffusion gradient for ABA uptake is systemically reduced. Different locations of *AtABCG22*, *AtABCG25 *and *AtABCG40 *in *Arabidopsis *plants, together with their effect on ABA transportation suggest that an efficient active transport for ABA movement is essential for plants to respond to a mass transport-based ABA signal under stress condition. Large number of the ABC transporter family and functional redundancy also suggest that plants employ a variety of ABC transporters for ABA transportation in response to different environmental cues (Antoni *et al*., 2011). Investigations on ABA transporter will, therefore, be of great importance in the field of ABA signaling. A unified nomenclature for *Arabidopsis *ABA transporter has recently been set up, which have provided much-needed clarity and a framework for future research (Verrier *et al*., 2008). In rice, no ABC transported has been reported to be an ABA transport to date. A comparison of exemplar tree showing phylogenetic relationships of Arabidopsis and rice proteins in ABC transporter has been done in the nomenclature, which will be helpful for searching the ABA transporter in rice.

## ABA receptors

After decades of intensively study on ABA, a large number of components in ABA signaling pathway have been identified. The recently identified ABA receptors are the most inspiring results among the research in this field (Shen *et al*., 2006; Liu *et al*., 2007; Park *et al*., 2009; Ma *et al*., 2009; Santiago *et al*., 2009; Kline *et al*., 2010). Although different opinions of these receptors have been raised (Johnston *et al*., 2007; Liu *et al*., 2007; Risk *et al*., 2008, 2009), the PYR/PYL/RCAR receptor seems to be the most acceptable one, which is isolated by four separately groups via various methods (Ma *et al*., 2009; Park *et al*., 2009; Santiago *et al*., 2009; Nishimura *et al*., 2010). After receiving ABA from the ABC membrane transporter, the PYR/PYL/RCAR-ABA complex will then inactivate the PP2Cs protein, negative regulators of the ABA signal. PYR/PYL/RCAR belongs to a SART-domain superfamily (Iyer *et al*., 2001). Structure analysis show an ABA binding site locates in the central hydrophobic cavities of PYR1, PYL1, and PYL2 (Melcher *et al*., 2009; Miyazono *et al*., 2009; Yin *et al*., 2009). The binding site is blanked with two loops, the gate and latch. Binding with ABA will induce the conformational change of the two loops, make them more closed, which in turn release an interaction surface for PP2C proteins. After combining with the START protein, a try from the PP2C protein contacts the ketone from ABA in a water-mediated manner. The formation of a ternary complex, PYR/PYL/RCAR-ABA-PP2C, will then relieve the negative input into the signaling pathway provided by the PP2Cs which inactivate the SNF1-related protein kinases (SnRK2s), a critical component of ABA signaling pathway.

**Figure 1:**
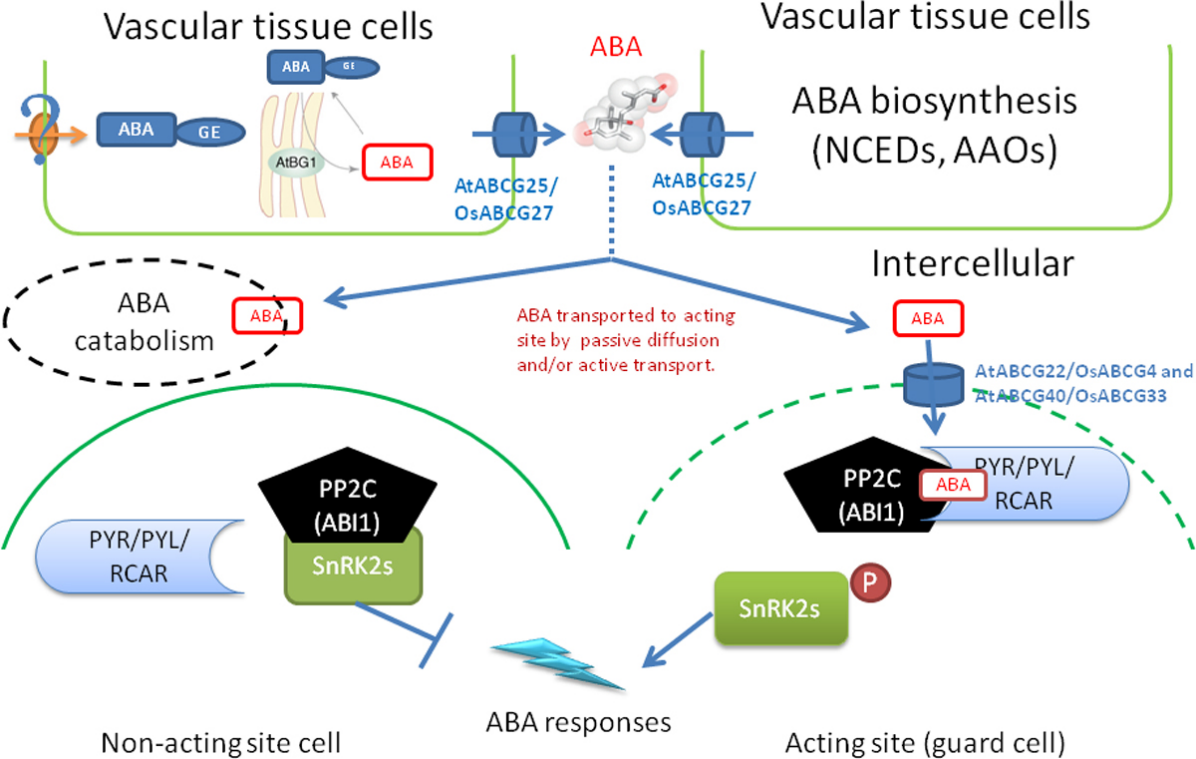
**A simplified model for abscisic acid generation, transport and perception in plants**. ABA biosynthesis is induced by environmental cues in the vascular tissues. ABA can also be released at ER from ABA-GE that stored in the vacuole or transported from the vacuolar system by an unknown ABA-GE transporter (? stand for unknown a mechanism). Both sourced ABAs are transported to intercellular space by the ABA transporters, AtABCG25 which majorly located in vascular tissue and exports ABA outward the cells. The newly synthesized ABA is then transported to the cells of acting site for ABA-responses by passive and/or active transport. The ABA importers and exporters can efficiently and directly deliver ABA to the acting site. However, part of the ABA is catabolic inactivated, which also plays a pivotal in regulating the intensity of ABA signal. PYR/PYL/RCAR receptors percept ABA signal intracellularly then combine with the negative regulators of ABA signal, PP2Cs/ABI1, to form a ternary complex. Thus, the negative regulator is inactivated whereas the downstream targets of PP2Cs, SnRK2s, are allowed to be activated by phosphorylation. The activation of SnRK2s will thus initiate an ABA response. In cell without ABA signal, the SnRK2s is inactivated by PP2Cs.

Further investigations of PYR/PYL protein have shown that the entire family (except for PYL13) is capable of activating ABA-signaling in response to ABA (Fujii *et al*., 2009). Their results therefore confirm the role of PYR/PYL protein as ABA receptors. Moreover, other research shows that some of the PYR/PYL proteins have preference on binding to different ABA format. Such as PYL9 chooses natural (+)-stereoisomer whereas PYL5 binds to (-)-ABA (Santiago *et al*., 2009). The binding preference of these receptors greatly increases the complexity of ABA signaling pathway. However, in other words, it is the extreme complexity that makes ABA one of the most critical massager in stress response for plants. Together with the powerful active transport system, as well as the downstream phosphorylation events, ABA receptors lead to a rapid, efficient, complicated and multi-stresses-responded signaling pathway (Figure 1).

## Biological roles of ABA in plants

Ever since the discovery of ABA in 1960s, many critical aspects of ABA's physiological functions have been established shortly afterwards. The phytohormone was proved to be involved in a number of physiological processes, such as synthesis of seed storage proteins and lipids, the promotion of seed desiccation tolerance and dormancy, and the inhibition of the phase transitions from embryonic to germinative growth and from vegetative to reproductive growth (Leung and Giraudat, 1998; Rock, 2000). Whereas in vegetative tissues, ABA is found to increase significantly in response to different environmental cues, like drought, salt, cold and so on (Shinozaki and Yamaguchi-Shinozaki, 2000). A dynamic accumulation of ABA in response to water stress has been well studied in maize (Ren *et al*., 2006) and rice (Zhu *et al*., 2009; Ye *et al*., 2011). Unlike other plant hormones, the endogenous concentration of ABA can increase more than 10-fold within a few hours of drought stress and decrease dramatically to normal levels following rehydration (Kushiro *et al*., 2004). The elevated ABA content is beneficial for plant under stress conditions in spite of the inhibition effect on plant growth. The water loss is reduced because of stomata closure induced by ABA under osmotic stress and because canopy expansion is reduced. Besides, many stress responsive genes which are favorable for biosynthesis of compatible osmolytes and LEA-like proteins are induced by ABA, thus prevent plants from stress damage and increase plant stress tolerance (Bray, 2002; Finkelstein *et al*., 2002). In contrast, the level of ABA, which is a negative regulator of shoot elongation, is decreased rapidly and thus results in an advanced elongation of shoot in submerged rice, one of the few crops that can temporarily survive under complete submergence (Sauter, 2000; Voesenek *et al*., 2003). *OsABA8ox1 *is proved to play a key role in mediating the decrease of ABA content in submerged rice, which is regulated by ethylene (Saika *et al*., 2007). The frequent utilization of ABA as a message transducer seems that plants do not have many plans to cope with the environmental stimulations. By contraries, the properties of ABA, such as variety of action sites, rapid turnover and transport, crosstalk with numerous signaling pathways and large number of downstream targets, have make it an extremely powerful message transducer in both normal and stress conditions. Comparisons of transcriptomes for *Arabidopsis *and rice exposed to ABA, drought, salt and other abiotic stresses have revealed that 5-10% of the transcriptome is changed by these treatments (Nakashima *et al*., 2009). This number in *Arabidopsis *is two to six times as many genes as are regulated by most of the other plant hormones (Nemhauser *et al*., 2006), The ABA-induced genes are enriched for those encoding proteins involved in stress tolerance while ABA-repressed gene products are enriched for proteins associated with growth. All the date indicates the central role of ABA in plants tolerance to abiotic stresses (Culter *et al*., 2010). Researches on roles of ABA in plants response to abiotic stresses has been the major focus in the field of ABA study. However, little has been done on the aspect of biotic stress-related functions of ABA. Thus, here we want to highlight the recent works on role of ABA in plant defense to biotic stresses.

The role of ABA in plant defense to pathogen attack has been well reviewed by Ton et al. (2009). Plant defense can be divided into three phases. They are: pre-invasive defense barrier (Phase I), early post-invasive defense (Phase II) and late defense (Phase III). Evidences have shown that ABA plays as a defense barrier to stop the invasion of pathogen by inducing stomatal closure (Melotto *et al*., 2006). Application of pathogen-associated molecular patterns (PAMPs), such as the flagellin derivative flg22 and lipopolysaccharides can induce the closure of stomata. However, such application does not have any effect on the stomata of ABA-deficient mutant *aba3-1 *plants, indicating the involvement of ABA in this defense response.

In the second phase of defense, which is characterized by rapid deposition of callose-rich cell wall enforcements and generation of reactive oxygen species (ROS), roles of ABA seem to be controversial and vary among different plant-pathogen interactions (Ton *et al*., 2009). In the bacteria invasion, ABA inhibits the induction of callose, evidenced by four mutants, the ABA hypersensitive mutant *aba1*, *aba2 *and ABA insensitive mutant *aba1-1*, *aba2-1*. The former two mutants deposit less callose whereas the insensitive mutant deposit augmented levels of callose (de Torres-Zabala *et al*., 2007). However, in plants invaded by oomycetes and fungi, ABA seems to suppress the ROS production which plays a key role in plant defense system (Asselbergh *et al*., 2007). ABA can also antagonizes the late SA-dependent resistance to pathogen attack (Yasuda *et al*., 2008) and interplay with JA to promote plants' susceptibility to pathogen (Anderson *et al*., 2004) in the late defense (Phase III). Although our knowledge about ABA's role in plant defense to pathogen is insufficient and contradictory results are sometimes reported, we still can discern a general pattern, in which ABA plays a positive role in early defense of plant by preventing pathogen invasion but a mostly negative influence at later colonization stages by a complex manner (Ton *et al*., 2009).

## Determinants of ABA signal intensity

As was mentioned above, the ABA content will not be increased significantly until the turgor of leave is zero although the soil has start to dry. However, plants under such condition have been detected closure of stomata, indicating ABA is synthesized in root and transported to guard cells through the xylem (Ren *et al*., 2007). In grapevine plant, which is far higher than the cereal plants, the root-sourced ABA signal is gradually intensified along the vine under both water-stressed and non-stressed conditions. Further experiments reveal that it is the pH gradient along the vine that plays a role in modifying and enhancing ABA signal, which is exhibited by more severely inhibiting of stomata conductance in higher leaves of the vine (Li *et al*., 2011). However, by using two ABA-sensitive promoters fused to the reporter gene β-glucuronidase or luciferase, Christmann et al. (2004) has found that the increase of reporter activities peak between 10 h and 14 h from the onset of water stress. Although the active ABA pool is generated in shoot but not in root in plant exposed to water stress for 24 h, their results indicate that both root-sourced and leaf synthesized ABA are required for ABA response in plant under water stress.

When the soil drying goes further to severely affect the leaf turgor, ABA will be dramatically increased by biosynthesis and deconjugation to initiate complex responses to different stresses. Indeed, the expressions of ABA biosynthesis genes *NCEDs*, *AAOs *and *ABA2 *in the vascular tissues, are significantly induced by stress conditions (Cheng *et al*., 2002; Tan *et al*., 2003; Koiwai *et al*., 2004). Besides, ABA-GE is efficiently deconjugated by *AtBG1 *in the endoplasmic reticulum (ER) system in *Arabidopsis *exposed to water stress (Lee *et al*., 2006). The ABA content inside the cell of *atbg1 *mutant plant is similar to the control plant whereas the extracellular ABA level of mutant plant is significantly lower than control plant, indicating that ABA is transported to the intercellular space after released from ABA-GE (Figure 1). Owing to these two effective origins for ABA production, the ABA signal can reach to the intensity which is necessary for initiating an ABA response. However, one thing should not be ignored is that ABA catabolism also plays a key role in contributing to the ABA signal intensity. In maize, the rate of ABA catabolism is found 11 times higher in plants exposed to water stress than normal condition (Ren *et al*., 2006). The recently identified ABA transporters suggest that ABA movement is not passive but active, indicating another limit factor for ABA signal intensity.

From the above discussion, we can found that it is because of the multi-determinants for ABA signal intensity, ABA is made one of the most critical signal massagers in plant signal network. However, little is known about the working pattern and relationship among these determinants in controlling the ABA signaling. Recent years, several investigations have revealed that plant will utilize various sets of determinants to cope with different stress conditions. In maize leaves under water stress, the stress ABA catabolic rate is 11 times higher than that in control plants. Pharmacological experiments proved that ABA biosynthesis in root required the xanthophyll precursors transported from leaves (Ren *et al*., 2006), indicating that an accelerated catabolic rate of ABA is favorable for providing the law materials for de novo synthesis of ABA in root and that root is critical in positioning ABA for rapid early adaptive responses in condition when ABA is needed. Their results indicate that although ABA biosynthesis plays a dominant role, ABA catabolism is also rapid enough to play an important role in the regulation of ABA accumulation. In consistent with them, the catabolism key gene *OsABA8ox1 *is also induced by water stress in rice (Ye *et al*., 2011). Glucose can delay seed germination, which is attributed, at list in part, to the increase of expression of ABA biosynthesis genes in Arabidopsis (Cheng *et al*., 2002; Chen *et al*., 2006). In rice, application of glucose does not have any effect on ABA biosynthesis but significantly represses the catabolism of ABA (Zhu *et al*., 2009). These results indicate that glucose delay seed germination by perturbing different determinants in monocotyledon and dicotyledon plants. Identification of *AtBG1 *gene, which is a β-glucosidase and identified to hydrolyze ABA-GE to produce bioactive ABA, reveals an even more fast mechanism in increasing ABA content. *atbg1 *mutant has similar but more slight ABA-deficient phenotype. The enzyme activity of AtBG1 by polymerization is responsible for the diurnal change of ABA level. Both of them are thought to be the two possible reasons why plant rely on *AtBG1 *for ABA rapidly production in response to stress conditions (Lee *et al*., 2006). The recently identified ABA transporters are also important regulator of ABA signaling intensity. By directly transport ABA to the action site, these transporters are also involved in determining the ABA level in the action site of ABA. Loss-of-function mutant of these mutants also display an ABA-deficient phenotype (Kang *et al*., 2010; Kuromori *et al*., 2010, 2011). All these investigations suggest a very complex model for controlling ABA level in the acting site. However, knowledge about the model is far from enough. The identification of ABA receptors and transporters has greatly completed our knowledge about ABA signaling pathway. Based on these findings, following work on ABA signaling will be much easier.

## Conclusion

Despite significant effort and progress that has been made over the last several decades, there are still many unknowns about ABA signaling pathway. Research on identifying new ABA receptors and constructing the working model of these receptors will still be the focus in the field of ABA investigation. The identification of ABA transporters has drawn our attention to the determinant of ABA signaling intensity. Plants have utilized different patterns of ABA signaling intensity determinants to cope with various stress conditions. Thus, it will be the further interest to study how these determinants cooperate with each other in controlling the intensity of ABA signaling.

## Competing interests

The authors declare that they have no competing interests.

## Authors' contributions

NY drafted the manuscript and participated in revising the manuscript. JL provided some suggestions to the manuscript. JZ conceived of the ideas of the article and revised the manuscript. All authors read and approved the final manuscript.
